# Morphometry of the lateral orbitofrontal cortex is associated with eating dispositions in early adolescence: findings from a large population-based study

**DOI:** 10.1093/scan/nsab084

**Published:** 2021-07-14

**Authors:** Peter A Hall, John R Best, James Danckert, Elliott A Beaton, Jessica A Lee

**Affiliations:** School of Public Health Sciences, University of Waterloo, Waterloo, ON N2L 3G1, Canada; Gerontology Research Centre, Simon Fraser University, Burnaby, BC V5A 1S6, Canada; Department of Psychology, University of Waterloo, Waterloo, ON N2L 3G1, Canada; Department of Psychology, University of New Orleans, New Orleans, LA 70148, USA; Department of Psychology, University of Waterloo, Waterloo, ON N2L 3G1, Canada

**Keywords:** eating, OFC, MRI, adolescence, diet, cortex

## Abstract

Early adolescence is a critical period for eating behaviors as children gain autonomy around food choice and peer influences increase in potency. From a neurodevelopmental perspective, significant structural changes take place in the prefrontal cortex during this time, including the orbitofrontal cortex (OFC), which is involved in socially contextualized decision-making. We examined the morphological features of the OFC in relation to food choice in a sample of 10 309 early adolescent children from the Adolescent Brain and Cognitive Development Study. Structural parameters of the OFC and insula were examined for relationships with two important aspects of food choice: limiting the consumption of fast/fried food and maximizing the consumption of nutritious foods. Raw, partially adjusted and fully adjusted models were evaluated. Findings revealed that a larger surface area of the lateral OFC was associated with higher odds of limiting fast/fried food consumption in raw [odds ratio (OR) = 1.07, confidence interval (CI): 1.02, 1.12, *P *= 0.002, *P*_FDR_* *= 0.012], partially adjusted (OR* *= 1.11, CI: 1.03, 1.19, *P *= 0.004, *P*_FDR_ = 0.024) and fully adjusted models (OR = 1.11, CI: 1.03, 1.19, *P *= 0.006, *P*_FDR_ = 0.036). In contrast, a larger insula volume was associated with lower odds of maximizing healthy foods in raw (OR = 0.94, CI: 0.91, 0.97, *P* <0.001, *P*_FDR_ = 0.003) and partially adjusted (OR = 0.93, CI: 0.88, 0.98, *P *= 0.008, *P*_FDR_ = 0.048) models. These findings refine our understanding of the OFC as a network node implicated in socially mediated eating behaviors.

## INTRODUCTION

The orbitofrontal cortex (OFC) receives information about the taste, smell, sight and texture of food and represents this in terms of its reward value in the medial OFC, areas 13 and 11 (Grabenhorst and Rolls, 2011; Levy and Glimcher, 2012; Rolls, 2016a, 2016b, 2021; Padoa-Schioppa and Conen, 2017; Hirokawa *et al.*, 2019). The OFC represents many other types of reward-related information as well, including that of a social nature (Rolls *et al.*, 1994, 2006; Hornak *et al.*, 1996, 2003; Rolls, 2019). In contrast, the lateral OFC responds to many aversive, subjectively unpleasant or unexpected events (O’Doherty *et al.*, 2001; Kringelbach and Rolls, 2003; Rolls *et al.*, 2003, 2020; Deng *et al.*, 2017; Xie *et al.*, 2021). Importantly, the lateral OFC is directly involved in reversal, in that damage to this region impairs the reversal or stopping of a behavior and increases impulsivity (Rolls *et al.*, 1994, 2020b; Berlin *et al.*, 2004; Hornak *et al.*, 2004; Aron *et al.*, 2014). Research on adolescent brain development (see Dahl, 2004 for a review) suggests that emotional and reward-related reactivity develops earlier than stopping abilities in the adolescent brain, meaning that adolescence is a time where subtle variations in the medial or lateral OFC, respectively, may have substantial implications for eating of foods that require self-control to avoid (e.g. calorie-dense fast or fried food items) and, to a lesser extent, the selection of nutritious foods that may be less hedonically appealing.

## Early adolescence, eating and the OFC

In adolescence, the prefrontal cortex (PFC) undergoes significant morphological changes and coincident with these are equally noteworthy changes in the social environment (Luna *et al.*, 2001; Larsen and Luna, 2018). From late childhood to early adolescence, the social network becomes broader and more impactful in many areas of function (Christie and Viner, 2005; Curtis, 2015). Eating behaviors and the social environment of eating both change considerably as youth gain autonomy around food choices, particularly with respect to accessing calorie-dense food items, which might be available both within and outside of the household. Amplifying such dynamics further is the newfound emotional significance of food choice, which can generate strong feelings of not only affiliation, acceptance and pleasure but also shame, disgust and guilt. As such, brain structures that connect socioemotional decision-making with homeostatic needs may be quite consequential for developing eating habits.

Prior studies examining the structural aspects of the human brain and eating outcomes have shown that body mass index (BMI) is negatively associated with gray matter volume (GMV) in the PFC, insular cortex and other medial structures, including the amygdala (Bobb *et al.*, 2014; Debette *et al.*, 2014). Other studies have revealed significant associations between volumetric parameters and obesogenic behaviors, such as inactivity and eating practices (Erickson *et al.*, 2010; Gu *et al.*, 2015; Papenberg *et al.*, 2016). Finally, a recent meta-analysis of 21 studies (with a cumulative total of 5885 participants) found that a lower GMV in the medial PFC (including the OFC) is one of the most reliable correlates of obesity (García-García *et al.*, 2019; Chen *et al.*, 2020). A recent analysis of the Adolescent Brain and Cognitive Development (ABCD) Study examined BMI (but not eating behaviors) in relation to functional and morphological aspects of the brain, using whole brain analyses (Adise *et al.*, 2021). Findings suggested widespread structural brain features associated with weight gain and baseline BMI, such as cortical surface area, thickness and subcortical volume. Two earlier studies using a smaller subset of the findings found similar morphological associations with BMI, most centrally, reduced cortical thickness in the PFC (Ronan *et al.*, 2020; Laurent *et al.*, 2020).

## Prior studies on OFC structure and eating patterns

Only a handful of prior studies have examined associations between brain structural parameters and eating patterns. In one of these (Song *et al.*, 2019), it was found that unrestrained eating was negatively correlated with GMV of the cingulate cortex, in a small sample (*n *= 159) of Chinese women. In a larger sample (*n *= 629) of young and middle-aged adults, self-reported hedonic eating symptoms were associated with larger right lateral OFC thickness over and above BMI; no other neuroanatomical correlates of food addiction were found (Beyer *et al.,* 2019). Finally, comparing overweight and obese to normal-weight participants in a case–control study (*n *= 139), Cohen and colleagues found that a greater OFC volume predicted more high-quality food choice tendencies recorded in a 3-day food diary among overweight and obese individuals, although medial and lateral subregions of the OFC were combined in this case (Cohen *et al.*, 2011). Functional imaging studies have similarly implicated the OFC in food cue reactivity and subjective hunger states (van der Laan *et al.*, 2011; Tang *et al.*, 2012; Chen and Zeffiro, 2020).

## The current investigation

Although several empirical studies and one meta-analysis have confirmed reliable associations between OFC structural parameters and BMI, relatively few studies have focussed on eating patterns that might give rise to BMI. Likewise, most prior studies of this nature have included large proportions of middle-aged and older adults, wherein brain structural parameters might represent the consequence of decades of adiposity and associated physiological states (e.g. hypertension and impaired glycemic control). For this reason, studies of OFC structural parameters in earlier life might be particularly informative. The ABCD is a large-scale, multi-center study of adolescent brain development and cognition (Jernigan *et al.*, 2018) and provides a unique opportunity to examine such effects. This study has recruited more than 11 000 9- and 10-year-olds to date, all of whom have undergone assessment of lifestyle behaviors (including eating habits) and brain imaging, the latter including a structural magnetic resonance imaging (MRI) scan.

The purpose of the current investigation is to examine the association between structural aspects of the OFC, its subregions and eating tendencies in a large sample of young adolescents. It is hypothesized, based on prior studies, that a larger OFC volume or surface area will predict more adaptive eating patterns across demographic features of the sample, because of the higher capacity to perform complex value computation from multiple value sources (including relative health value, hedonic appeal and approval of others). Second, it is anticipated that such effects will be mediated by measures of value computation (e.g. delay discounting) and emotional regulation capacity. Finally, it is hypothesized that morphological aspects of the insula will be associated with eating tendencies, but with less consistency, due to lower loading of function on value computations that may influence choice behavior itself, and more encoding of salience. Such effects will be particularly evident with respect to calorie-dense food items and will remain after controlling for demographic characteristics, methodological variables and BMI.

## Methods

### Participants

The ABCD Study is a large-scale prospective study following cohorts of 9- and 10-year-olds forward in time over the adolescent years into adulthood, with a projected 10-year time horizon (Jernigan *et al.*, 2018; https://abcdstudy.org/). The current investigation involves the first two waves of data collection, which includes a baseline MRI scan (Wave 1), and assessments of eating habits (Wave 2; corresponding to ages 11 and 12 years). Participant demographic characteristics are presented in Table 1. The sample consists of all individuals with baseline MRI scan and Wave 2 parent-reported eating habits (*N *= 10 309). The ABCD Study involved a consortium of 21 data collection sites in major metropolitan areas across the continental United States. The original recruited sample comprises 11 880 participants from Data Release 2.0.1, aged between 9 and 10 years of age at baseline (Wave 1). Baseline visits occurred between September 2016 and October 2018.

**Table 1.** Descriptive statistics (*N* = 10 309^a^)

Characteristic	
Age in months, baseline [mean (s.d.)]	119.0 (7.5)
Age in months, follow-up [mean (s.d.)]	131.1 (7.7)
Sex	
Female	4931 (48)
Male	5378 (52)
Child race	
American Indian/Native American	57 (0.6)
Asian Indian	47 (0.5)
Black/African American	1576 (15)
Chinese	80 (0.8)
Don’t know	73 (0.7)
Filipino	47 (0.5)
Guamanian	1 (<0.1)
Japanese	11 (0.1)
Korean	21 (0.2)
Native Hawaiian	3 (<0.1)
Other Asian	26 (0.3)
Other Pacific Islander	13 (0.1)
Other race	431 (4.2)
Refused	44 (0.4)
Samoan	3 (<0.1)
Vietnamese	20 (0.2)
White	7834 (76)
Missing	22
Child Hispanic ethnicity	
Yes	2038 (20)
No	8148 (80)
Missing	123
Family income level [mean (s.d.)]	7.3 (2.4)
Missing	806
Primary parent education, years [mean (s.d.)]	16.7 (2.7)
Missing	11
Second parent/partner education, years [mean (s.d.)]	16.5 (3.0)
Missing	2006
Healthy food consumption (whole grains, green leafy veggies, other veggies and berries)	
0 (none of these categories)	490 (4.8)
1 (1 of these categories)	1432 (14)
2 (2 of these categories)	2760 (27)
3 (3 of these categories)	3224 (31)
4 (all of these categories)	2403 (23)
Fast/fried food consumption less than once per week	6820 (66)
Body mass index *z*-score, baseline [mean (s.d.)]	0.5 (1.8)
Missing	761
Body mass index *z*-score, follow-up [mean (s.d.)]	0.9 (1.8)
Missing	1152
Emotional Stroop congruent mean reaction time (ms) [mean (s.d.)]	1087.9 (125.0)
Missing	146
Emotional Stroop incongruent mean reaction time (ms) [mean (s.d.)]	1164.1 (136.0)
Missing	150
Delay discounting (area under the hyperbolic discounting curve) [mean (s.d.)]	0.5 (0.3)
Missing	149
Lateral orbitofrontal cortex, thickness (mm) [mean (s.d.)]	3.0 (0.1)
Lateral orbitofrontal cortex, area (mm^2^) [mean (s.d.)]	5572.3 (598.1)
Lateral orbitofrontal cortex, volume (mm^3^) [mean (s.d.)]	18 669.2 (2032.8)
Medial orbitofrontal cortex, thickness (mm) [mean (s.d.)]	2.7 (0.2)
Medial orbitofrontal cortex, area (mm^2^) [mean (s.d.)]	3710.6 (427.2)
Medial orbitofrontal cortex, volume (mm^3^) [mean (s.d.)]	11 974.8 (1441.9)
Insula, thickness (mm) [mean (s.d.)]	3.3 (0.1)
Insula, area (mm^2^) [mean (s.d.)]	4545.4 (490.8)
Insula, volume (mm^3^) [mean (s.d.)]	15 200.6 (1692.0)
Cerebral cortex, mean thickness (mm) [mean (s.d.)]	2.8 (0.1)
Cerebral cortex, total area (mm^2^) [mean (s.d.)]	1 86 269.7 (18 098.0)
Cerebral cortex, total volume (mm^3^) [mean (s.d.)]	5 96 059.6 (56 700.3)

a Values are expressed as *n* (%), unless otherwise indicated.

Each individual ABCD Study site obtained local institutional ethical review board approval, and centralized institutional approval for the ABCD Study was obtained by the University of California, San Diego. For each participating child, written and informed consent was provided by each caregiver; each child provided written assent. The current analysis was approved by the ethical review board of the institution of the lead author (the University of Waterloo, Waterloo, Canada).

### Procedures

#### MRI and preprocessing.

The ABCD consortium performed the neuroimaging and preprocessing. Images of brain structure were acquired using MRI. Cortical volume, area and thickness were estimated using Freesurfer v5.3.0 (Dale *et al.*, 1999). Images were subjected to manual checking by technicians to evaluate the image quality (intensity inhomogeneity, underestimation of white matter, pial overestimation and magnetic susceptibility artifact). Those assigned a score of 1 for passable image quality were used for the current investigation. Further details regarding quality control procedures are described in Hagler *et al.* (2019). Brain morphological features (area, volume and thickness) for the OFC and insula were obtained from automated anatomical parcellation using the Desikan atlas–based classification (Desikan *et al.*, 2006) as part of the standard FreeSurfer pipeline [region of interest (ROI) names in the atlas: lh-insula, rh-insula, lh-medialorbitofrontal, rh-medialorbitofrontal, lh-lateralorbitofrontal, rh-lateralorbitofrontal]. For each structure, left and right hemispheres were combined, and in the case of the OFC, medial and lateral subregions were examined separately.

### Measures

#### Anthropometrics.

To assess BMI, separate weight and height measurements were taken and averaged together; from this average score, BMI (zBMI) was calculated using corresponding *z*-scores according to the World Health Organization Child Growth Standards (World Health Organization, 2006). This was done using the R package ‘zscorer’ (Myatt and Guevarra, 2019). BMI *z*-scores <5 were assumed to be invalid and recoded as missing.

#### Demographics.

Household income was collected via caregiver report using the following categories: $5000, $5000–$11 999, $12 000–$15 999, $16 000–$24 999, $25 000–$34 999, $35 000–$49 999, $50 000–$74 999, $75 000–$99 999, $100 000–$199 999 and ≥$200 000. Prior to being included in the statistical models, income was categorized into tertiles (<$50 000 *vs* $50 000–$99 999 *vs* $100,000 and above). Sex-at-birth was reported at baseline by parents in accordance with sex listed on the child’s birth certificate; all subsequent analyses used this as the indicator of participant sex. Child race/ethnicity was reported by parents at this time as well.

#### Delay discounting and emotional regulation.

The negative and positive urgency sub-scales from the Urgency Perseverance Premeditation Sensation seeking UPPS-P for Children Short Form (ABCD-version) were collected at baseline and were included as measures of emotion regulation (Barch *et al.*, 2018). Delay discounting is a behavioral measure of impulsive decision-making (Johnson *et al.*, 2008) and was collected at Wave 2. The participant makes several choices between a small-immediate hypothetical reward right now and a constant hypothetical $100 reward at different future time points (6 h, 1 day, 1 week, 1 month, 3 months, 1 year and 5 years). Each block of choices features the same delay to the larger reward, and the immediate reward is titrated after each choice until both the smaller-sooner reward and the delayed-$100 reward have equal subjective value to the participant. An ‘indifference point’ is calculated that represents the small-immediate amount deemed to have the same subjective value as the $100 delayed reward at each of the seven delay intervals. These values were then converted to an area under the hyperbolic curve, which quantifies the degree of discounting of delayed rewards (Myerson *et al.*, 2001).

#### Emotional Stroop task.

This task is an adaptation of the classic Stroop paradigm (Stroop, 1935), which is intended to measure cognitive control with emotionally salient stimuli (Başgöze *et al.*, 2015; Banich *et al.*, 2019). Stimuli consist of pairs of face images and emotion words denoting positive and negative emotional states, which can be either congruent with each other or incongruent with each other, in terms of valence (the word ‘happy’ paired with a happy face image; the word ‘sad’ paired with a happy face image). The location of the word varied from trial to trial. Two test blocks were completed: the first included 75% congruent and 25% incongruent trials; the second block included 50% congruent and 50% incongruent trials. Accuracy and reaction time are recorded, and longer reaction times on incongruent relative to congruent trials were the metric of interference (i.e. low cognitive control). For the current study, we used the mean response time on incongruent trials across block and emotion subtype, adjusted for congruent response time, as the outcome measure.

#### Eating behaviors.

At 1-year follow-up, the primary parent completed a nutrition questionnaire, which included 13 questions on food categories eaten framed as follows: ‘In a typical week, does your child eat…’ (Morris *et al.*, 2015). For the current study, we constructed two outcome measures from responses to this questionnaire. First, we used the response to the item regarding consumption of fried or fast food less than once per week as a binary outcome. Second, we summed the responses across four items (greater than three servings of whole grains per day, greater than six servings of green leafy vegetables per week, at least one serving of other vegetables per day and at least two servings of berries per week) to create a healthy food variable that ranged from 0 (none of the healthy item servings met) to 4 (all of the healthy item servings met). As such, both eating behavior metrics were scored such that responding ‘yes’ indicates a healthier food choice in relation to the target food type(s) (i.e. ‘yes’ = limiting fast and fried food; ‘yes’= maximizing servings of vegetables, berries and whole grains). Several items from this complete ABCD parent-report measure involving protein sources were omitted because of their less definitive roles in relation to healthy eating outcomes and/or confounding with vegetarian status (e.g. processed meat products).

### Statistical analyses

All statistical analyses were conducted using R version 4.0.3 (r-project.org). To evaluate the association between baseline structural MRI markers and 1-year nutritional outcomes, two sets of regression models were constructed. Consumption of fast and fried foods less than once a week (yes *vs* no) was modeled using logistic regression, whereas consumption of healthy foods (including whole grains, green leafy vegetables, other vegetables and berries) was modeled using ordinal regression. For both sets of regression models, each of the a priori structural brain measures were evaluated without covariates and then with covariates. In the covariate-adjusted models, the primary covariates were baseline child age, sex, race and ethnicity, family income and total cortical volume, total cortical area or mean cortical thickness, depending on whether the primary brain structure predictor represented volume, area or thickness; zBMI was examined as an additional covariate in a third set of models. As such, each outcome was examined in raw (Model 1), covariate-adjusted (Model 2) and covariate + zBMI adjusted (Model 3) predictive models. Given the substantial sex differences often observed in research involving eating and disproportionate social pressure around eating for adolescent girls, we also examined sex as a moderator. Brain variables were standardized (mean = 0, s.d. = 1) to facilitate interpretation and comparison across brain variables. Estimates from these models were converted to odds ratios (necessitated by the binomial eating predictor variables, as collected by the ABCD Study), which are presented along with the 95% confidence intervals (CIs) and *P* values. Corrections for false discovery rate (FDR) were applied across three brain regions (i.e. lateral OFC, medial OFC and insula) and both morphological outcomes (i.e. surface area and volume), as per Benjamini and Hochberg (2000). Statistical significance was set at *P*_FDR_ < 0.05 for each estimate of interest.

Secondary analyses employed linear regression to determine whether structural MRI parameters were associated with performance and measures of delay discounting and emotional regulation. These included the negative and positive urgency scales from the UPPS, the delay discounting area under the curve and the emotional Stroop incongruent response time adjusted for congruent response time. Models were constructed without covariates and then with the same set of covariates included in the primary models.

## Results

### Primary analyses

**Fig. 1.**
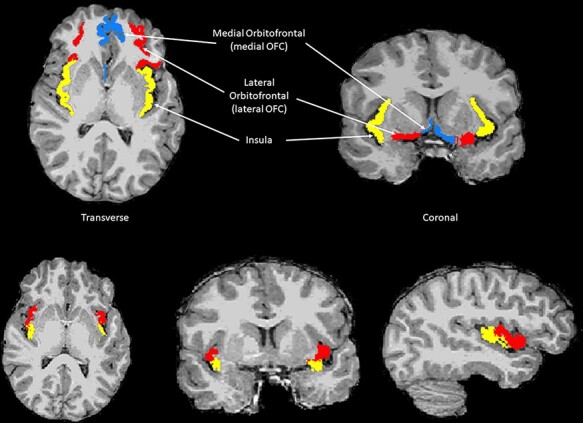
ROIs, including the medial and lateral OFC, as well as the insula.

**Fig. 2.**
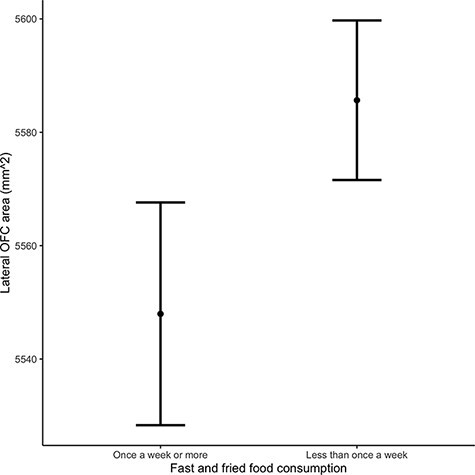
Difference in lateral OFC surface area by fast-food consumption. Error bars represent 95% CIs.

**Fig. 3.**
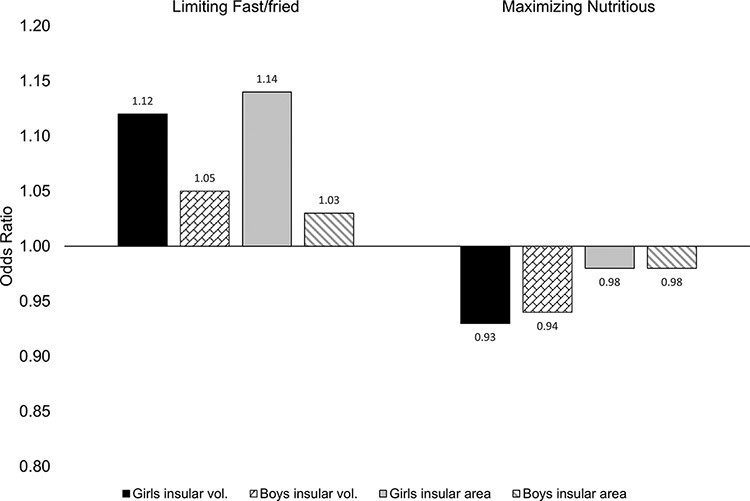
Sex differences in insular morphology predicting odds of limiting fast/fried food consumption and maximizing nutritious food consumption.

Descriptive statistics for the sample (*N *= 10 309) can be found in Table 1. Analysis of structural parameters and eating habits is presented in Table 2, in raw and adjusted forms. Findings from raw and partially adjusted models revealed that larger lateral OFC surface area was associated with higher odds of limited fast and fried food consumption [adjusted odds ratio (OR) = 1.11, 95% CI: 1.03, 1.19, *P*_FDR_ = 0.024]; this effect remained strong following further adjustment for BMI. Medial OFC was not observed to be associated with eating habits, with all FDR-corrected *P* values >0.20. Larger insula volume was associated with a lower odds of maximizing nutritious foods (adjusted OR = 0.93, 95% CI: 0.88, 0.98, *P*_FDR_ = 0.048), although this did not survive further adjustment for BMI. Figures 1 and 2 depict the morphological substrates for each ROI and the primary findings regarding OFC area, respectively. Effect sizes (Hedge’s g) predicting odds of limiting fast/fried food consumption and maximizing nutritious foods are depicted in Figure 3, for the lateral and medial OFC subregions and for the insula.

**Table 2.** Summary of regression models involving orbitofrontal cortex and insula as predictors of eating tendencies among adolescents

	Limiting fast/fried				Maximizing nutritious (greens/fruit/grains)			
Primary predictor	OR	95% CI	*P*	*P*_FDR_	OR	95% CI	*P*	*P*_FDR_
OFC (lateral)								
Volume								
Unadjusted	**1.06**	**1.01, 1.10**	**0.013**	**0.039**	1.03	0.98, 1.09	0.260	0.260
Adjusted^a^	1.05	0.97, 1.12	0.200	0.300	1.03	0.97, 1.10	0.290	0.310
Adjusted^a^ + zBMI	1.04	0.97, 1.12	0.296	0.355	1.03	0.96, 1.09	0.410	0.430
Area								
Unadjusted	**1.07**	**1.02, 1.12**	**0.002**	**0.012**	1.04	0.98, 1.10	0.240	0.260
Adjusted^a^	**1.11**	**1.03, 1.19**	**0.004**	**0.024**	1.05	0.99, 1.11	0.100	0.300
Adjusted^a^ + zBMI	**1.11**	**1.03, 1.19**	**0.006**	**0.036**	1.05	0.99, 1.12	0.120	0.300
OFC (medial)								
Volume								
Unadjusted	1.01	0.97, 1.06	0.600	0.600	1.05	1.00, 1.11	0.0730	0.146
Adjusted^a^	0.97	0.91, 1.03	0.300	0.360	1.03	0.98, 1.08	0.310	0.310
Adjusted^a^ + zBMI	0.96	0.90, 1.02	0.232	0.348	1.03	0.97, 1.08	0.310	0.430
Area								
Unadjusted	1.03	0.99, 1.08	0.200	0.240	1.04	0.98, 1.10	0.190	0.260
Adjusted^a^	1.02	0.95, 1.09	0.600	0.600	1.04	0.98, 1.10	0.170	0.310
Adjusted^a^ + zBMI	1.02	0.95, 1.10	0.589	0.589	1.05	0.98, 1.11	0.150	0.300
Insula								
Volume								
Unadjusted	**1.05**	**1.01, 1.10**	**0.020**	**0.040**	**0.94**	**0.91, 0.97**	**<0.0010**	**0.003**
Adjusted^a^	**1.07**	**1.00, 1.15**	**0.039**	0.078	**0.93**	**0.88, 0.98**	**0.0080**	**0.048**
Adjusted^a^ + zBMI	**1.08**	**1.00, 1.15**	**0.038**	0.076	**0.94**	**0.88, 0.99**	**0.0270**	0.162
Area								
Unadjusted	**1.05**	**1.00, 1.09**	**0.036**	0.054	**0.94**	**0.91, 0.98**	**0.0010**	**0.003**
Adjusted^a^	**1.07**	**1.01, 1.14**	**0.035**	0.078	0.97	0.92, 1.02	0.240	0.310
Adjusted^a^ + zBMI	**1.08**	**1.01, 1.15**	**0.031**	0.076	0.98	0.92, 1.03	0.430	0.430

OR = odds ratio for a 1 s.d. increase in the predictor. ORs > 1 suggest that an increase in the structural measure is associated with a more positive nutrition outcome (i.e. more limiting of consumption of fast/fried foods or more maximizing consumption of nutritious foods).

Bold values are significant after correction for false discovery rate.

a Adjusted for child age at the time of MRI, sex, race, Hispanic/Latino ethnicity, family annual income, data collection site at baseline and total cortical volume, total cortical area or mean cortical thickness, depending on the particular structural brain predictor in the analysis.

Analysis of the left and right hemispheres separately revealed that both left and right lateral OFC areas were associated with greater odds of limiting fast/fried food consumption (adjusted OR = 1.09, 95% CI: 1.03, 1.17 and adjusted OR = 1.07, 95% CI: 1.01, 1.15, respectively). Similarly, the negative association between insula volume and healthy food consumption was observed in the left hemisphere (adjusted OR = 0.94, 95% CI: 0.89, 1.00) and right hemisphere (adjusted OR = 0.94, 95% CI: 0.89, 0.99). See Supplemental Table 1 for details.

### Secondary analyses

The association between lateral OFC area with delay discounting and emotional Stroop performance is shown in Table 3 and with negative and positive urgency in Table 4. After accounting for the covariates, the lateral OFC area was not significantly associated with any of these measures. We further considered whether income tertile, sex or age might moderate the association between brain predictor and nutritional outcome; however, we failed to detect any significant interactions despite substantial statistical power (all *P*s > 0.05), suggesting that the associations between these brain regions and nutrition outcomes are similar for males and females and across family income. Sex differences were examined as well, given the large difference in eating expectations in the American context for girls *vs* boys. None of the tests of sex-based moderation of OFC effects were significant, suggesting that OFC effects on eating were relatively uniform for girls and boys in the sample. However, insula surface area was significantly more impactful on fast/fried food consumption for girls (OR* *= 1.14, CI: 1.05, 1.25) than for boys (OR = 1.03, CI: 0.95, 1.12; χ^2^ (1) = 4.08, *P *= 0.043); ORs for each sex category and outcome variable are presented in Figure 3. No additional sex differences emerged with respect to insula volume.

**Table 3.** Summary of regression models involving lateral OFC area and performance measures of inhibitory control and delay discounting measured at Wave 2

	Delay discounting (area under the curve)			Emotional Stroop incongruent condition		
Primary predictor	Standardized estimate	95% CI	*P*-value	Standardized estimate	95% CI	*P*-value
OFC (lateral) area						
Unadjusted	−0.01	−0.03, 0.01	0.27	−0.01	−0.02, 0.002	0.14
Adjusted^a^	0.01	−0.02, 0.05	0.42	0.003	−0.01, 0.02	0.71

a Adjusted for child age at the time of MRI, sex, race, Hispanic/Latino ethnicity, family annual income and total cortical area. Additionally, emotional Stroop incongruent scores are adjusted for congruent condition scores.

**Table 4.** Summary of regression models involving lateral OFC area and youth-reported measures of emotional control

	UPPS negative urgency			UPPS positive urgency		
Primary predictor	Standardized estimate	95% CI	*P*-value	Standardized estimate	95% CI	*P*-value
OFC (lateral) area						
Unadjusted	−0.03	−0.05, −0.01	0.006	−0.05	−0.07, −0.03	<0.001
Adjusted^a^	−0.02	−0.06, 0.01	0.14	−0.004	−0.04, 0.03	0.84

a Adjusted for child age at the time of MRI, sex, race, Hispanic/Latino ethnicity, family annual income and total cortical area.

## Discussion

In this investigation, it was found that a larger surface area of the lateral OFC was associated with stronger tendencies to limit consumption of appetitive but unhealthy calorie-dense foods (i.e. fast/fried foods) among early adolescent boys and girls. This effect remained robust after accounting for demographics, methodological variables and BMI. The absolute magnitude of the effect was modest but reliable after stringent correction for FDR. As such, there was reasonable support for our primary hypothesis. Inconsistent with our primary hypotheses, there was no evidence of a mediation of the abovementioned effects by delay discounting task performance or a self-reported measure of emotional control. With respect to the insula, larger surface area and volume predicted a stronger tendency to limit fast/fried foods, and this association was significantly greater in magnitude for girls than for boys. On the other hand, a larger insular surface area was associated with weaker tendencies to maximize nutritious foods. Our findings are consistent with other animal and human research implicating the OFC in eating and food processing more generally (Simmons *et al.*, 2005; Spierling *et al.*, 2020; Rolls, 2021), as well as body mass (Chen *et al.*, 2020), although the medial OFC is sometimes implicated more so than lateral (Shott *et al.*, 2015).

Given that the lateral OFC is involved in changing or stopping behavior when punishment or non-reward stimuli are received (Rolls, 2019; Rolls *et al.*, 2020a), it is hypothesized that the larger surface of the lateral OFC may encode a greater sensitivity to the non-rewarding or punishing attributes of high-calorie food indulgence, such as social sanctions and norm violations. Such contingencies may be furnished by peer groups—which are disproportionately influential at this time—or even in the home environment, by siblings, parents or others in the home.

The effects of the insula are also notable, given the involvement of the insula in salience detection (anterior insula; Craig, 2009) and mobilization of organismic responses to physiological states of homeostatic significance (posterior insula; Menon and Uddin, 2010). In general, the foods included in the vegetables/berries/grains item were nutritious but of low caloric density, meaning that a physiological drive toward calorie maximization would tend to result in lower odds of endorsing this item; indeed, that is what was found. The parcellation employed did not allow for separate consideration of anterior and posterior aspects of the insula, unfortunately; future studies may further disentangle the relative role of each. Nonetheless, it is noteworthy that pathologies involving overactive insular function may involve ascription of undue emotional valence to mundane or unimportant stimuli, as we might see in anxiety disorders and the personality disposition of neuroticism; there is some potential that insular involvement in eating may work through a similar mechanism (i.e. hyper-emotional significance of food objects, rendered salient in the everyday environment). The home environment and parental eating behaviors may be implicated in this respect as well, given that much of the eating activity in early adolescence is still in the family home. The parents also provide access to different food types, and so the home food cue array is partially modulated by parents.

Some of the strengths of this investigation included a large and broadly representative sample, which allowed for the possibility of subregion analysis within the OFC and highly precise estimates of most estimates of association. Although other studies have identified the OFC as an important structure in the prediction of BMI, to our knowledge none have previously examined medial and lateral subregions separately and with eating behaviors as the target. The consideration of these separately appears to be somewhat consequential, given that lateral subregions seemed to predict eating tendencies more reliably than medial OFC subregions. Additionally, the precise parameter estimates allow us to confidently declare null findings to be definitively null. Finally, the population-representative nature of the sample allows for generalizability to the US adolescent population.

The selection of participants on the cusp of adolescence (9–10 years) is a strength in that it reduces the impact of lifelong adiposity on the brain, as an alternative explanation for why associations are observed. Most existing studies documenting associations between brain structural parameters and BMI, for instance, have employed samples wherein the majority of participants are in middle age to late life, meaning that obesity status is maintained for decades prior to measurement of brain structure or function. Any effects of obesity on the brain may influence correlations between structural parameters and predictors of interest, including eating behaviors. In this case, the majority of the sample is not yet obese, and those who have significant adiposity have likely had it only for months to years, rather than years to decades. This makes us more confident that brain structural parameters represent premorbid facets of the brain that may predict future adiposity, via eating behaviors.

Some limitations exist. First, longitudinal (i.e. multi-year) follow-ups might be more informative in terms of confirming temporal associations between OFC characteristic emergence and later eating trajectories; although the MRI data were collected at baseline and eating behaviors at follow-up, directionality cannot be conclusively disentangled without further follow-up measurements. Second, the effects observed, strictly speaking, are not large in magnitude. However, given that the OFC is thought to only participate in value computation and comparison, it stands to reason that feed forward to lateral PFC subregions would be required for motor implementation (i.e. actual implementation of a value preference in manifest eating behaviors; Padoa-Schioppa and Conen, 2017). From this perspective, the magnitude of the OFC effects observed here should be as expected. With respect to measurement, the eating behavior measures were parental reports, which may have eliminated self-presentational biases, which can be substantial among adolescents. However, parental reports may have also introduced inaccuracy in estimation, given that parents do not have full access to the dietary history of their children, even over the course of a given time interval. Ultimately, these influences would tend to attenuate the strength of correlations between eating measures and structural parameters, however. With respect to null findings, some null effects may be produced by a lack of sensitivity of the binomial eating outcome, which may have also attenuated any potential mediating effects involving behavioral measures tested in this investigation.

Further, although our focus in this review was on brain systems that model some facets of social context in eating decisions (and therefore a focus on the OFC), we did not include in our ROI analyses subcortical structures that might mediate indulgent eating behaviors, such as the nucleus accumbens and ventral tegmental area (Volkow *et al.*, 2011). Investigating these areas may represent a useful future direction. Likewise, patterns of interconnectivity of these and the current ROIs may be useful in this respect, and the insula in particular, as it has many theoretically meaningful connections of significance to eating-related pathologies (Frank *et al.*, 2013). Finally, the morphological features of the insula extracted for the current investigation are not isomorphic with the primary taste cortex, given that the taste cortex is only a small part of the anterior insula. Unfortunately, the atlas used for parcellation in ABCD data does not include the subregions of the insula. As such, it is the case that the relationship between the insula and any eating habits is as likely to reflect somatic awareness and any other insular functions over and above taste *per se*. Broadly speaking, our findings fit well with an interpretation of insular function as involving salience detection in any case.

## Conclusion

In conclusion, we examined the morphological aspects of the OFC and insula and their associations with several aspects of eating behaviors among early adolescents in a large, representative population dataset. We found that a larger lateral OFC surface area reliably predicted less frequent indulgence in calorie-dense food items (fried or fast foods). In contrast, a larger insula volume was associated with less consumption of foods that are of high nutritive value but of relatively low-to-moderate calorie density. Our findings are broadly consistent with the idea that the lateral OFC regulates some aspects of indulgence, possibly in concert with increasing social pressures to regulate during the early adolescent years. A highly sensitive insular processing in contrast may render adolescents relatively more sensitive to highly salient foods rather than more nutritive options available in their eating environment.
